# Participant, peer and PEEP: considerations and strategies for involving people who have used illicit substances as assistants and advisors in research

**DOI:** 10.1186/s12889-018-5765-2

**Published:** 2018-07-05

**Authors:** Alissa M. Greer, Ashraf Amlani, Bernadette Pauly, Charlene Burmeister, Jane A. Buxton

**Affiliations:** 1 School of Population and Public Health, University of British Columbia, Vancouver, Canada; 2 BC Centre for Disease Control, University of British Columbia, Vancouver, Canada; 3 School of Nursing, Canadian Institute for Substance Use Research, University of Victoria, Victoria, Canada

**Keywords:** Community-based participatory research, Peer engagement, Research methods, Qualitative research, Participation, Drug use

## Abstract

**Background:**

The Peer Engagement and Evaluation Project (PEEP) aimed to engage, inspire, and learn from peer leaders who represented voices of people who use or have used illicit substances, through active membership on the ‘Peeps’ research team. Given the lack of critical reflection in the literature about the process of engaging people who have used illicit substances in participatory and community-based research processes, we provide a detailed description of how one project, PEEP, engaged peers in a province-wide research project.

**Methods:**

By applying the Peer Engagement Process Evaluation Framework, we critically analyze the intentions, strategies employed, and outcomes of the process utilized in the PEEP project and discuss the implications for capacity building and empowerment among the peer researchers. This process included: the formation of the PEEP team; capacity building; peer-facilitated data collection; collaborative data analysis; and, strengths-based approach to outputs.

**Results:**

Several lessons were learned from applying the Peer Engagement Process Evaluation Framework to the PEEP process. These lessons fall into themes of: recruiting and hiring; fair compensation; role and project expectations; communication; connection and collaboration; mentorship; and peer-facilitated research.

**Conclusion:**

This project offers a unique approach to engaging people who use illicit substances and demonstrates how participation is an important endeavor that improves the relevance, capacity, and quality of research. Lessons learned in this project can be applied to future community-based research with people who use illicit substances or other marginalized groups and/or participatory settings.

## Background

Community based participatory research (CBPR) is an increasingly popular methodology due to its potential to improve research relevance, validity, and reliability through the participation of the community the emphasis on action as part of the research process [1–5]. CBPR has been described as a collaborative approach to research that aims to democratize knowledge by involving community members in the research process itself and promotes social transformation rather than simply the generation of knowledge [3, 5]. Collaborative and equitable partnerships with community members can improve the relevance, acceptability, and effectiveness of research by ensuring the questions, approaches, and media for dissemination are appropriate [6, 7]. Involving community members in the research process can be seen as a strengths-based approach to building capacity among the individuals and communities studied [8].

People who use illicit substances (PWUS) who use that experience to inform their professional work, commonly referred to (herein) as ‘peers,’ are increasingly participating in research, practice, and policy processes. Involving peers in this work can democratize knowledge and promote social change when power and control are distributed equitably among team members [7, 9]. For peers, involvement in CBPR can improve confidence by validating their knowledge, expertise, and experience, and can decrease isolation and increase capacity [6]. Although there are international reports of CBPR where peers are members of the research team [10], documentation and reflection of the participatory research process with PWUS is relatively scant in the literature. One literature review noted this gap, highlighting the need for further exploration and documentation of the engagement of PWUS [11].

The roles of community members involved in the research process can vary widely in terms of responsibilities, decision-making power, and length or depth of involvement. Peers’ can be engaged as research partners or leaders, or be limited to roles similar to others involved in research (i.e. assistants), or participate as consultants in research advisory groups [6]. Each of these roles vary in the nature of control and power in decision making that is shared between peers and other team members [6]. To date, there has been little guidance on, or inquiry into, the application of each of these roles.

### Peer engagement in Canada and the PEEP project

Within Canada, peer engagement has been gaining traction as a best practice approach to designing health and harm reduction solutions and is widely endorsed in principle [11–14]. Researchers from the British Columbia Centre for Disease Control (BCCDC) and harm reduction stakeholders from the British Columbia Harm Reduction Strategies and Services (BCHRSS) Committee established a collective vision for the future of peer engagement in the province: PWUS should be hired into paid positions for meaningful work that values their lived experience, and be treated as equal to other employees doing similar work who do not identify (publicly) as a PWUS. Members of the BCHRSS emphasized the need for research and services collaborating with peers at all stages of the research process, along with recognition of peers’ contributions.

**Fig. 1:**
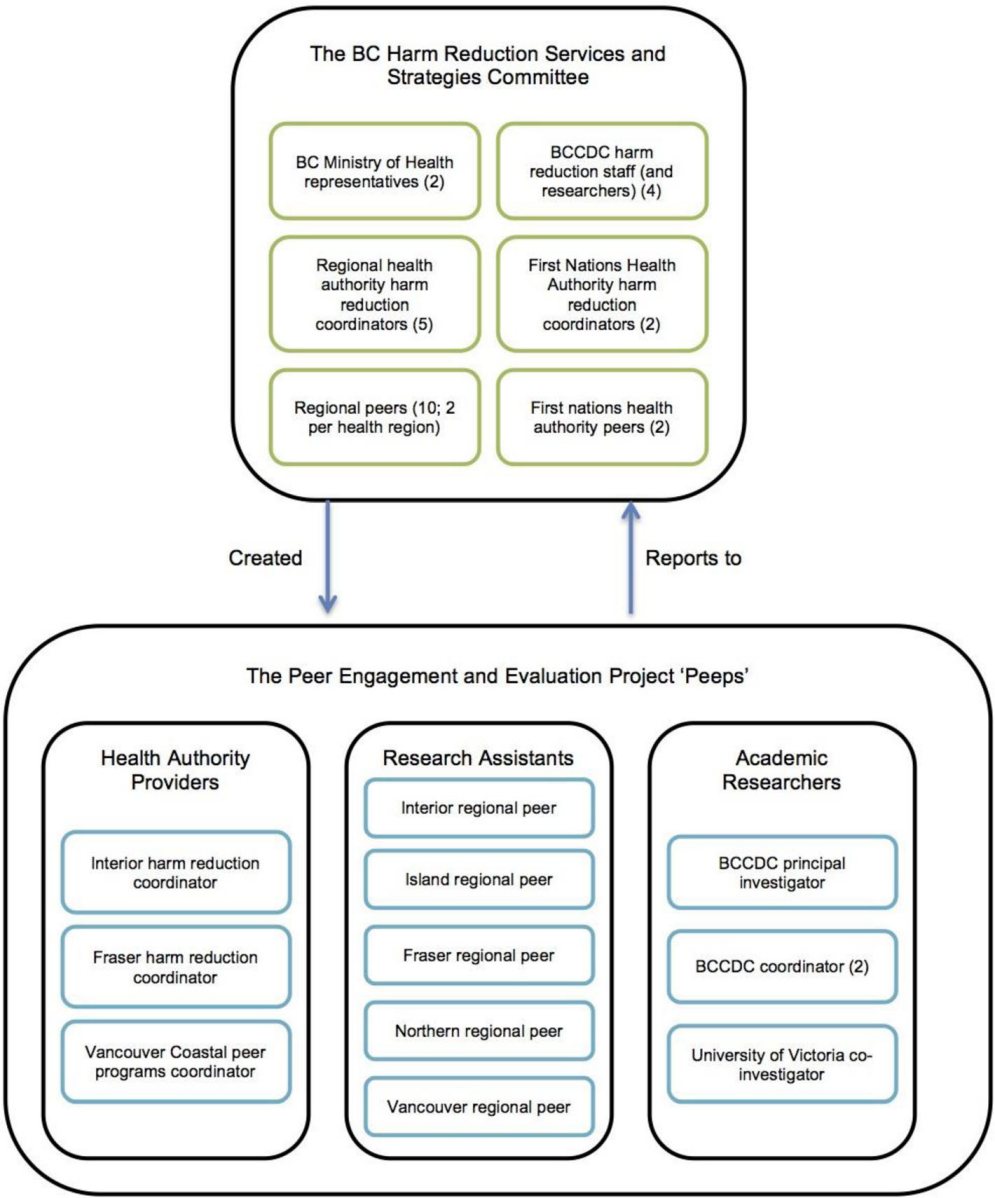
Organizational structure and make-up of the BC Harm Reduction Services and Strategies Committee and the Peer Engagement and Evaluation project

This identified need was the impetus for the creation of the Peer Engagement and Evaluation Project (PEEP) at the BCCDC [15]. The BCCDC oversees provincial programs which provide health promotion and prevention services, and policy support to government and health authorities to help reduce disease and preventable injury in BC. PEEP was a research project conceived of by the BCHRSS committee who consist of peers, service providers, and academic researchers (Fig. 1). Through the BCCDC and from the BCHRSS committee, PEEP sought to: 1) develop a peer network, 2) create and distribute peer engagement principles and best practices for provincial health authorities, and 3) engage with peer project leaders, which would ultimately bring a broader representation of peers’ voices.

**Fig. 2:**
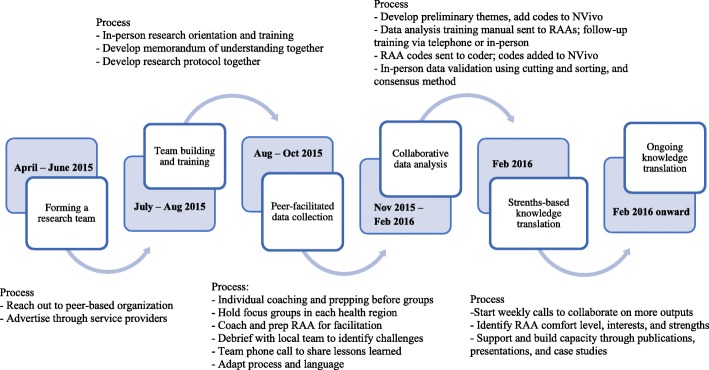
Peer Engagement and Evaluation Project process

The goal of this paper is to critically and reflexively evaluate the methodological and pragmatic considerations that arise when engaging peers in CBPR through reflections on the PEEP research process (Fig. 2). This paper, written in partnership with peers employed with PEEP, is structured as follows: First, we briefly present the PEEP participation process, which offers a rich description of one participation process that other CBPR researchers and community members alike may learn from. Second, in the spirit of reflexivity and evaluation, we assess this process using the Peer Engagement Process Evaluation Framework (Table 1) [16]. Third, we share some of the methodological and practical considerations that emerged from this evaluation. The strategies offered can be used in designing future participatory processes with the community, and in this case PWUS, as assistants and advisors in research contexts (Table 2). Lastly, we conclude with a discussion about the implication of our findings, critically reflecting on the evaluation and involving PWUS in CBPR.

**Table 1:** Evaluation of the PEEP process using the peer engagement process evaluation framework^a^

Goal	Description of evaluation criteria	Constructs of evidence	Evidence of progress or opportunity for improvement
Equitable participation	Ensure experiences are respected and represented to address the diverse health needs of each community.	• Diversity at the table • Power and distribution of voices at the table • Flexibility	• Engaging with peer-based organizations • Representatives from each region • Flexible commitment • Open communication
Supportive environment	Assess and address barriers and facilitators of engagement; ‘environment’ encompasses micro, meso, and macro levels.	• Community-building activities • Advanced planning • Structure of protocols	• A clear hiring process • Fair compensation • Clear role expectations
Capacity building and empowerment	Develop the abilities of individuals and groups defined in terms of access, ability, mobilization, interest, networks, opportunity, and literacy.	• Community building • Social capital, skills, confidence • Enhanced peer network	• Training remotely and in person • Various training mediums • Strengths-based approach to activities • Collaborative activities • Independent activities • Challenge of activity and capacity needed increasing over time • Knowledge of research and subject matter building over time • Team building activities
Peer-informed research^b^	The explicit and implicit evolution of the research in relation to the purpose identified; ability to understand local risk environment, synthesize information, and design relevant solutions.	• Informed the protocol development, analysis, and outputs	• Knowledge translation and sharing findings • Peer facilitated data collection • Collaborative data analysis • Collaborative and diverse knowledge translation

^a^Greer et al., 2016 [14]

^b^Framework goal adapted to fit a research setting

**Table 2:** Lessons and strategies for engaging people who use substances or peers as active members of a research project

Hiring and recruiting • Hiring over the duration of the project rather than a short, one-off commitment • Hire more than one peer within each region to promote support and representativeness • Engage with peer-based organizations wherever possible	
Fair compensation • Pay peers the same as others (who do not have substance use experience) in similar roles • Navigate institutional and structural barriers to compensation early on • Discuss barriers early on and develop solutions together • Be transparent and flexible with compensation	
• Lessons and strategies for engaging people who use substances Gather feedback or develop the job advertisement with peers, including the description of what the role entails • Develop a team memorandum of understanding early on; revisit this often	
Communication • Adapt communication to different learning styles and literacy • When possible, do training and other activities with peers in person	
Connection and collaboration • Meet often and consistently • Print and mail all materials if working remotely • Be flexible and committed; give space and support as participation shifts across time	
Mentorship • Hire a peer mentor early on who can help navigate the process and support others	
Peer-facilitated research • Ensure peers participate in as much decision making as possible, not just some decisions (or decisions that are convenient) • Ensure peers are involved and provide feedback on any materials that have been developed by others early on and throughout the process – beginning to end	

## Methods: The Peep process

### Building a team of Peeps

A diverse group of PWUS were sought to work as research assistants and advisors (RAAs) on the PEEP project. First, a description of the RAA position was developed and reviewed by peers engaged with other BCCDC projects. Then, BCCDC researchers approached peer-based organizations (in regions where they existed) or distributed the description to harm reduction service providers known to work with peers. The individuals hired represented a diversity of backgrounds, ethnicities, ages, and regions (i.e. rural and urban). This group of five peers (herein referred to as “the RAAs”) participated as peer researchers and the advisory committee for PEEP. The RAAs^1^ joined a team that included several people that did not identify (publicly) as PWUS. Together, the RAAs and non-peer researchers formed a dynamic team – herein referred to as “the *Peeps*”. The structure of the Peeps and their relation to the BCHRSS committee who initiated the project can be found in Fig. 1.

### Capacity building

To initiate the project, the Peeps came together for an interactive, three-day orientation and training in July 2015. The non-peer researchers delivered a visual and interactive “Research 101” training through PowerPoints, printed material in binders, and flip charts. The training covered basic research concepts and approaches, with a focus on qualitative research methods. The objectives of PEEP were reviewed and discussed in detail. The Peeps then developed more concrete research questions, a focus group recruitment strategy, question guides, and travel plans for their regions. The team often broke into smaller groups and discussed these strategies in greater detail, reporting back to the larger group.

During this meeting the Peeps developed a Memorandum of Understanding which aimed to establish expectations including project goals, team member goals, recognition of the personal risks of participation, responding unmet expectations or disagreement in perspectives, crediting members of the team, and disseminating the findings to community partners. This document also included how each Peep would contribute to decisions, the length and scope of the project, resources, training, support, communication, confidentiality and disclosure, compensation, and what happens at project end. This Memorandum was revisited annually.

### Peer-facilitated data collection

Following the training in July 2015, smaller groups of the Peep team (one RAA and two coordinators) traveled throughout each region of BC, holding focus groups with local PWUS. From July to October 2015, thirteen focus groups were held with 83 PWUS across rural and urban communities. In addition to the training provided at the July meeting, the RAAs were supported through individual coaching prior to the focus groups to ensure they felt prepared and comfortable in facilitating, and team members provided support during the focus groups where needed. Each focus group was followed by a debrief with the participating team members to discuss challenges and identify the emerging themes. Every two weeks during this time, the team also held a phone call to provide support to each other and to share their experiences. For instance, the RAAs found the question guide to be too wordy and included technical language which some PWUS did not understand. In keeping with the flexible nature of PEEP, the question guide was adapted to include language more acceptable to the community.

### Collaborative data analysis

To organize and focus the abundance of qualitative data collected, an independent data coder thematically coded the data in NVivo using three preliminary themes. These initial themes were inductively developed by three of the BCCDC non-peer researchers by working with the data coder and synthesized the focus group field notes, and read transcripts meeting minutes, and field notes. These themes were provisional as it was important to gather RAA input. Later, the themes were modified after discussing the input from RAAs (described herein).

Data analysis was an opportunity to build the RAAs research skills further. In the fall of 2015, the RAAs were sent a training manual and copies of a focus group transcript of which they had facilitated. Each manual described the goals and steps of qualitative analysis through visuals and plain language. This training was discussed during the team phone meetings as well as individually until the RAAs felt equipped. Codes that the RAAs developed were added to NVivo and used to revise the primary codes into four more specific themes. Several subthemes were highlighted and salient quotes the RAAs highlighted were extracted.

After this coding process, the entire team met again in Vancouver in February 2016. Here, one full day was spent validating data using a method adapted from Bogdan and Biklen’s ‘cut-up-and-put-in-folders’ approach [16]. Two teams were formed so that each could cover multiple themes, one after another. Subtheme headings were written on flip charts and pre-selected quotes were printed on individual pieces of paper. By consensus, the team decided what the quote referred to, which subtheme it belonged to, and pasted it below that heading. Some subthemes were changed, combined, or rejected. Often the meaning of quotes were discussed – a conversation led by the RAAs. These quotes made up the focus groups results, and were incorporated in all of PEEP’s outputs, including the Peer Engagement Principles and Best Practice Guidelines [12], PEEP infographic [17], and Compassionate Engagement modules [18].

### Strengths-based approach to outputs

The Peeps were encouraged and supported to participate in various knowledge translation activities. Participation varied per the level of comfort of each RAA. Some felt confident with public speaking, while others participated in community events or outreach locally. The Peeps shared their experiences and supported others on the team regularly, which was promoted through weekly or bi-weekly calls, as well as financial resources and training. To date, the RAAs and other Peeps have attended and presented at various conferences, co-created knowledge translation tools, and engaged in other policy and programming initiatives. The Peeps have also co-authored several community and academic publications and a series of case studies to engage health care providers to consider their own engagement practice.

## Methods: Evaluation methods

The Peer Engagement Process Evaluation Framework [16] used to evaluate the PEEP process was first published in *BMC Public Health* in 2016. The framework consists of four process goals: supportive environment; equitable participation; capacity building and empowerment; improved programming and policy [16]. The framework was originally developed to evaluate peer engagement initiatives in harm reduction and other health services and policies. However, we slightly modified the framework so it could apply specifically to a research context. To do so, we revised the goal of ‘improved programming and policy’ to ‘peer-informed research.’ This modified goal and evaluation criteria have been noted in the evaluation framework (Table 1).

The modified evaluation framework was used to qualitatively assess the PEEP process using several data sources including meeting minutes, field notes, and reflections of the first twenty months of the PEEP research project (February 2015 to September 2016). Meeting minutes were kept and shared among the Peeps from virtually every PEEP meeting (*n* = 29) during this time period. The field notes and reflections of all the Peeps during data collection and in writing this manuscript also served as important data. These data were first reviewed, then discussed in detail among co-authors (AG, AA, CB, BP, and JB). The process PEEP employed was written out in full (see PEEP process above) by co-authors and finally assessed against the evaluation framework (AG and AA). This collaborative process allowed us to both deductively (through the evaluation framework criteria) and inductively (by developing new categories and examples of constructs in the framework) identify strengths and opportunities from PEEPs approach.

## Results

Critical insights gained from evaluating the PEEP research process using the peer engagement process evaluation framework are outlined below. In addition, strategies that can be used to develop participatory processes in CBPR are shared (Table 2).

### Recruiting and hiring

Recruiting, hiring, and working with peers as paid members of the PEEP project offered unique opportunities and challenges that are worth reflecting on. In the initial planning stage of PEEP, we considered hiring several local peer recruiters on very short-term contracts. However, in reflecting on the participatory and emancipatory goals of PEEP, the decision was later made to hire five regional RAAs for the duration of the project (three years). In doing so, there was a stronger community voice in PEEP, making it more appropriate, relevant, and accessible to the community. The research benefited by obtaining more relevant and comprehensive data, and by creating an experience truer to the spirit and benefits of peer engagement. However, in reflection, the RAAs would have benefited from an additional RAA being located in their region, to support each other and provide better representation of PWUS from that area.

We also learned that hiring peers to represent their communities was particularly challenging in regions that have less access to harm reduction services. Previous research has identified that it is possible to successfully recruit peers into research through the internet [1]. The community of PWUS that we sought were a more hidden and disconnected group, particularly in rural areas such as in Northern British Columbia. Working with peer-based organizations allowed the community to nominate peers from their regions, which proved to be more accessible than recruiting peers through service providers. Discussions with the RAAs and peer-based organizations revealed that those from organizations were better connected, trained, and informed than peers recruited through non-peer service providers. These peers had the opportunity to ask questions and gain clarity on the job advertisement through their organization. Organizations also knew which peers were best suited and prepared for the work.

### Fair compensation

In PEEP, it was important that compensation was equitable and in line with participatory principles [14, 19, 20]. As such, the RAAs were paid the same wage ($25 CAD) as other team members who were paid hourly – namely, the research coordinators. Although this wage was higher than what peers are paid in similar work (locally), the RAAs provided feedback that the wage diffused power differentials, made them feel like valued members of the team, and that it reflected their expertise and skills they were offering to the project. In this regard, their lived experience was valued equivalent to graduate education.

However, paying peers was also the greatest challenge in hiring peers as RAAs. Specifically, the processes of navigating organizational procedures for payment with incessant bureaucratic barriers and delays. In attempting to provide a low barrier hiring process, the project coordinators faced several challenges in setting up employment contracts and paying RAAs, which resulted in late compensation of the work RAAs had completed. These delays were particularly pertinent among peers as they belong to an already marginalized group and were more affected by compensation delays than other team members. Delays in pay revealed unequal power dynamics between team members. Frustrations from these delays created an initial sense of distrust and stunted the relationships built between team members.

It also became clear that RAAs faced different challenges than the non-peer researchers in having to navigate institutional and financial structures. Some RAAs did not have consistent mailing addresses or bank accounts in which to deposit paycheques. Others faced barriers in navigating the local income assistance policies and earning limits. PWUS belong to a marginalized group that often have unique financial circumstances and barriers that should be discussed, addressed and resolved at the time of hiring peers. In reflection, the PEEP team has stressed that peers’ circumstances should not be assumed but discussed one-on-one. In addition to a guide for paying peers [21], a paper critically examining the issues at hand when compensating peers was written (under review).

### Role and project expectations

By reflecting on the recruitment process, we learned that recruiting PWUS is best when the process is peer-informed. Gathering substantial feedback from PWUS on the language used in the job advertisement as well as the way in which it is advertised (format, distribution) should be done in partnership with peers. Reflecting critically as a team, the RAAs voiced they also would have liked clear expectations of their roles. Some RAAs were unaware of the job advertisement and did not fully understand the scope of the project when they started.

To make roles and expectations clearer, the group developed a team Memorandum of Understanding in the first in-person meeting. However, we learned the document was too wordy and difficult to access for the RAAs as they would have to access to it online. The project would have benefited by creating an easily accessible, one- or two-page document that was revisited more frequently.

### Communication

In working remotely with the PEEP team, it became apparent that clear communication and support were paramount to our success. Communication adapted to varying learning styles, accessibility, interests, and skills. For instance, we learned that the lack of training materials after the in-person training in July did not equip the RAAs with the skills they needed to continue to succeed remotely. This gap was made evident while debriefing after the initial focus groups, and we learned that it was important to do more one-on-one training. Keeping the line of communication open with the group and taking the time to debrief was essential to getting this feedback and setting the RAAs up for success in the focus groups.

The importance of clear communication also became apparent during the qualitative coding training with RAAs. The training manual was mailed to the RAAs, and they trained remotely. The majority of the training was done independently although support was provided where needed. Feedback on this independent training was mixed, depending on the RAAs learning and collaboration style. The RAAs perceived the training as helpful among those who preferred hands-on learning, while others struggled with learning independently. The training guide itself was overwhelming for some RAAs to use on their own, while for others it was straightforward and clear. In reflection, the guide could be better used if RAAs had been paired with another RAA or academic researcher from their area from the beginning. Also, feedback regarding the accessibility of the document could have been gathered from peers, and peers could have been sent the audio recording from the focus group.

### Connection and collaboration

Running a cross-jurisdictional CBPR project was challenging as the team members worked remotely and often felt disconnected. Due to the province’s geography and budget limitations, most meetings occurred over the phone, leaving the team feeling disconnected at times. Face-to-face meetings worked much better than teleconferencing or collaborating online, as they allowed for team-building and avoided the problems posed by varying access to phone and Internet. We realized that a key component to our success when working remotely was regular telephone check-ins, and the ability to come together as a team in-person regularly. Originally, the telephone meetings were held every two weeks; however, after feedback from the RAAs about feeling disconnected after the focus groups, the meetings were held weekly. This regular connection became essential to work on many of the outputs that the Peeps collaboratively created, but also to ensure the Peeps felt supported, informed, and equipped with the tools they needed to succeed. The RAAs were also mailed documents in advance of discussions as there was challenges accessing computers and printing documents. Although only two or three times yearly, the in-person meetings were an opportunity to build safety and trust so that all team members felt comfortable taking risks, contributing their experience, and voicing concerns.

In efforts to stay connected while working remotely, the group adapted and learned to provide each other with professional and personal support. Both the RAAs and non-peer researchers faced several personal and professional crises throughout the project. It is important to note that these crises were not limited to those who identified as the peers on the project and were not explicitly substance related. Team members experienced issues related to serious illness or hospitalization of family members, overdoses among friends, evictions, and personal relationship problems. Like other CBPR, we found that each of us was able to participate at different times and in different ways that shifted based on the stability of our lives (Salmon et al., 2012). During periods of illness, housing or other issues, it was important not to assume people’s willingness or capacity to work on the project. We encouraged self-care, the practice of clear personal and professional boundaries, scheduled weekly check-ins, and allowed people to step away for a short time. By providing flexibility, giving space, respecting our differences, and working through crisis periods together we managed to build stronger relationships.

### Mentorship

Peer mentoring has been used as a health promotion strategy to build capacity and can be used within participatory research methodologies [22]. The idea and strategies for mentorship was introduced by the Peeps in February 2016 – nine months after the RAAs were hired. A peer mentor was hired to support the RAAs and PEEP project overall. However, the RAAs had grown personally and professionally throughout the project, and they were providing mentorship and support to each other, as well as to other peers in their community. PEEP would have benefited from hiring a peer mentor from the start. A mentor with lived experience of substance use *and* research could have informed the hiring and training process and supported the RAAs in different ways than the non-peer researchers.

### Peer-facilitated research

The RAAs were well situated to recruit and engage local peers in focus groups, creating a safe and familiar space, which increased the accuracy of data. In the debriefing sessions, the Peeps shared that the locations peers selected (i.e. shelters, harm reduction sites, peer organizations) were appropriate and contributed to feelings of a “safe space.” However, some communities where the RAA did not reside (despite being within the region of the RAA) were difficult to recruit from, and advertising, incentives (food, cash), accessibility, and transportation were sometimes overlooked in these locations. For instance, in one town, despite attempts to hold a focus group on two separate occasions, no participants attended. We came to realize that the nonresponse from the community was primarily due to the lack of in-person peer-based recruitment beforehand as the RAA was not from that community and the Peeps did not spend time in advance to identify and connect with PWUS there. This experience highlights the importance of working with local peers to inform all aspects of the research process, including local recruitment.

The RAAs also informed the question guide so that the content, language, and outcomes were relevant and understandable to other PWUS. The question guide initially developed mainly by the academic researchers with little input by the RAAs. Although we had broken into small groups to develop the guide further, it was early in the project and the capacity of all team members, as well as power dynamics and trust, were barriers to meaningful participation. As such, the initial question guide was not successful in eliciting responses in the first focus group. However, the RAAs were able to provide feedback after they had seen how it performed, and work as a team to edit and adapt the guide to be more culturally and linguistically appropriate for using with groups of PWUS. Through their active involvement in data collection and validation, we reduced potential bias and false assumptions in data interpretation, ensuring the results reflected the realities within their communities.

## Discussion

The Peer Engagement and Evaluation Project (PEEP)—a research project aimed to establish peer engagement best practice guidelines for service providers—empowered peers and other researchers as active members of the Peeps. We have described the process of working with peers who were employed as RAAs (research assistants and advisors). With a focus on building capacity over the course of the project, the RAAs played a main role in developing the research protocol, conducting and leading focus groups, coding, analyzing and interpreting data, and leading knowledge translation activities. By building research capacity within communities of PWUS, rather than simply involving peers in discreet aspects of the research, the Peeps grew professionally, while giving a voice to peers in multiple settings, and overall contributing to the empowerment of PWUS across the province and elsewhere.

Researchers are often urged to reflect critically on both the purpose and methods of the research to encourage the recognition of power that is related to knowledge and the production of that knowledge [23]. Given that peer engagement in research and at policy tables is relatively new both locally and internationally, learning from PEEP and other projects that have involved peers will be key to developing future initiatives that are effective and relevant to the communities they engage. As in most participatory work, the PEEP project was a flexible and iterative process that required ongoing reflexivity and critical reflection. Reflexivity offers a strategy to embrace collaboration, ongoing consultation and negotiation, and opportunity for capacity building and empowerment among communities of PWUS [23].

Previous research has pointed out that CBPR often brings together two “different worlds… [with] conflicting understanding of what constitutes legitimate research, competing agendas, and power inequities” [24]. CBPR requires careful attention to address such power dynamics during the research process [24]. Involving PWUS who belong to, generally, a marginalized and stigmatized group in society [25] introducing power, oppression, and resistance to team dynamics which are supported and reinforced through individuals, organizations, and structures that we work with [26].

The roles and relationships of the peers and providers expanded beyond our expectations as the project unfolded. First, the capacity and tenacity of the RAAs on the project was far greater than anticipated. This was particularly evident during periods of crisis and the ability of the group to adapt. We learned that we each face and struggle with crises in our lives. In other work settings, colleagues may not disclose challenges occurring outside the work place, but for us it was important in order to support where each of us were at (both personally and professionally) in the project at different times. Also, our learning was reciprocal – peer and non-peer researchers grew throughout the project. Communication, support, and reflexivity were key to our growth. We learned that in this work it is important to have a safe space to share with others – whether that be with other peers or staff who can support them both professionally and personally. By using reflexivity individually and as a team throughout the project, we continually recognized, understood, and adapted our dynamics and expectations. This process diffused power dynamics and made for a more meaningful experience overall.

These experiences highlight the transformative nature of CBPR in terms of social change and social justice, which have broader social implications that are embedded within the wider context of the marginalization and stigmatization of PWUS. As a marginalized and stigmatized group [25], PWUS are often excluded from policy forums, program development, and research that ulimately affects their lives [26]. Meaningfully and equitably involving peers in these processes can mitigate stigma and power differences [14, 26, 27]. We revealed several strategies for engaging PWUS which often relate to complex intersecting stigmas, structures and barriers that marginalized groups face. We recognize that peers are a heterogenous group with varying social and structural positions depending on the context [28]. Many PWUS are housed, literate, financially included, and exercise resistance and advocacy in the face marginalization and oppression. We also recognize the issues and strategies discussed here could apply to other groups, such as people living with mental illness.

Our findings add to a small but growing body of literature that examines the roles of PWUS in CBPR. Past research has focused on the ethics of engaging PWUS, which has problematized and drew attention to how participation can be disempowering or exclusionary [20, 29–31]. Along with issues of representation, compensation, capacity building, and pragmatics [29], discussions have engaged with questions of if communities can contest forms of social stigma that are often reproduced in these settings [20, 31], but perhaps only when researchers are supported in ways that address and reduce power heirarchies [31]. One way to do this is by engaging with peer-based organizations who can help navigate these heirarchies and support peer researchers [31], or community advisory committees [32]. Others have suggested that CBPR researchers must redefine their conceptualizations of ‘success’ in a CBPR project – “reaching a balance between ideal and realistic forms of community participation can be especially tricky in cases of CBPR with active drug users” [33]. In fact, as authors point out, the literature on partipation often views it as a continuum, which takes on a iterative, fluid approach that cannot be based on clear ‘success’ or ‘failure’ terms, and may work for differently for each person [33].

While PEEP was participatory in many aspects, it would not be considered ‘true’ CBPR. We had engaged the community before PEEP was funded by doing interviews and focus groups to identify issues that were important. However, as seen in other CBPR, this information gathering process and grant writing stage was not participatory as resources available were limited [34]. The PEEP project emerged as a response to the BCHRSS’s need for guidance on how to meaningfully engage peers in harm reduction settings. In any participatory research, partners may have a different set of priorities that do not align with or do not precede research grants [35]. Unfortunately, there is an inherent challenge in CBPR when applying for and receiving funds *before* engaging with the community. Participants may have other priorities and interests than those described in funding applications, which can cause frustration and conflict amongst the team. Conversely, there are ethical concerns in asking for support and increasing expectations of the community by developing funding proposals with them which later may not be granted. Alternatively, rather than engaging with the community as part of discrete projects, researchers could forge ongoing relationships and engagement as a process that develops with the community or peer-based organizations over time [36]– an approach where these relationships can better facilitate involvement and input from the time of conception.

Many CBPR projects use a community advisory committee that oversees all aspects of the research [32]. These roles are important to distinguish, as the role of a peer as a co-researcher on a project operates at a different level in the partnership power gradient than as an advisor [37]. In PEEP, rather than co-researchers or a ‘true’ community advisory committee, the RAA role was more to advise on decisions and direction in the research that was initially determined by the scope of the research grant (and non-peer researchers). These constraints were partially dictated by the resources available and expectations of the funder, but initially created unequal power dynamics between the RAAs and non-peer researchers. However, despite these constraints, over time power and decision making diffused across the Peeps, making it a democratic process. Although community advisory committee members functioning as partners is ideal, in reality members are often placed in the role of assistants or consultants due to challenges including logistics, finding partners, commitment, training, and timing of the project [37]. One area for opportunity may be to offer peers a dual role as a member of an advisory committee as well as peer researchers or knowledge brokers [36]. PEEP offers one model where peers co-created *and* advised much of the research process alongside other researchers. By examining this model, we add to the conversation and support involvement of peers in CBPR [6].

PEEP performed other aspects of CBPR. In theory, CBPR has been described as an “orientation to research” that focuses on relationships between the Peeps, with goals of societal or social change rather than a using specific set of techniques [24]. CBPR rests on two “pillars”: giving back to the community that research takes from, and community empowerment [35]. The Peeps developed a strong sense of trust, collaboration, and ownership within the team *and* the community. As well, the amount of reciprocal learning and growth was profound; we saw tremendous personal and professional growth among the RAAs and other researchers alike. Furthermore, the outcomes of the PEEP project have community benefit – the peer engagement best practice guidelines will promote the inclusion of PWUS internationally. In these ways, PEEP has held its integrity in its participatory process.

The lessons in this paper are not limited to research settings; the practices of engaging with peers can be applied to other community settings such as in drug policy and service provision. Internationally, peer involvement has been incorporated in a range of projects in the drugs research and policy field. For instance, several projects have focused on user involvement in treatment settings in Australia [38, 39] or the drugs policy research field [26, 40]. These projects have gathered information qualitatively [26, 40], quantitatively [32, 41, 42], and others have emphasized the ethics of involving peers in research [10, 32, 41, 42]. The roles of peers in CBPR projects in the past have varied, but all have stressed that seeking and incorporating the perspectives of PWUS can play an important role counterbalancing discriminatory opinions.

Several limitations to this paper should be acknowledged. We reviewed evidence from internal meeting minutes, field notes, and reflections from the Peeps against the Peer Engagement Process Evaluation Framework [16]. However, primary pre- and post-evaluation data from peers was not collected. Peer engagement and research projects have benefited from a formal evaluation of their process [43], including baseline measures of the team’s capacity and involvement. Where possible, this evaluation should be designed within a participatory framework. Despite no formal evaluation of PEEP, we reviewed records of the process and outcomes of our work, and the Peeps approved and supported this evaluation, its findings, and what is reported in this manuscript.

## Conclusion

By critically reflecting on the process of engaging the peers as research assistants and advisors to the research, we have shown that participation of the community in research is an important and worthy endeavour that improves the relevance, capacity, and quality of the research. The PEEP project successfully supported capacity development across all members of the Peeps, and empowered PWUS to take on leadership roles. By recruiting and fostering a team of peers, we have enriched the validity and applicability of the PEEP project outcomes and have ensured that this research process will promote change. The methods developed in this project provide important insights for future researchers engaging PWUS in their work and provides an example of how one might engage peers in future qualitative and participatory research projects.

## Abbreviations

BCCDC: British Columbia Centre for Disease Control
BCHRSS: British Columbia Harm Reduction Services and Strategies
CAD: Canadian dollar
CBPR: Community-based participatory research
PEEP: Peer Engagement and Evaluation Project
PWUS: People who use illicit substances
RAA: Research assistant and advisor

## Footnotes

1 After discussing the term “peer,” the team decided to remove from the RA job title (and this paper) as the team felt it may result in being treated differently than those identified as non-using members of the team. Rather, the word “peer” is only used in the context of highlighting the experiential knowledge that is contributed through this role.

## References

[1] Barratt MJ, Lenton S (2010). Beyond recruitment? Participatory online research with people who use drugs. Int J Internet Res Ethics.

[2] Funk A, Van Borek N, Taylor D, Grewal P, Tzemis D, Buxton JA (2012). Climbing the “ladder of participation”: engaging experiential youth in a participatory research project. Can J Public Health.

[3] Leung MW, Yen IH, Minkler M (2004). Community based participatory research: a promising approach for increasing epidemiology’s relevance in the 21st century. Int J Epidemiol.

[4] Salmon A, Browne AJ, Pederson A (2010). ‘Now we call it research’: participatory health research involving marginalized women who use drugs. Nurs Inq.

[5] Wallerstein NB, Duran B (2006). Using community-based participatory research to address health disparities. Health Promot Pract.

[6] Guta A, Flicker S, Travers R, Switzer S, Bungay V, Husbands W, et al. HIV CBR Ethics Fact Sheet #1: Ethical issues related to compensation [Internet]. Toronto, ON; 2014. Available from: http://www.hivethicscbr.com/documents/HIVCBREthics_FactSheet01.pdf. Accessed 30 June 2018.

[7] Israel BA, Schulz AJ, Parker EA, Becker AB (1998). Review of community-based research: assessing partnership approaches to improve public health. Annu Rev Public Health.

[8] Minkler M (2004). Ethical challenges for the “outside” researcher in community-based participatory research. Health Educ Behav.

[9] Roche B, Guta A, Flicker S (2010). Peer research in action I: models of practice [Internet].

[10] Crabtree A. It’s powerful to gather: a community-driven study of drug users’ and illicit drinkers’ priorities for harm reduction and health promotion in British Columbia, Canada [Internet] [Doctor of Philosophy]. [Vancouver, BC]: University of British Columbia; 2015 [cited 2015 Dec 28]. Available from: https://open.library.ubc.ca/cIRcle/collections/ubctheses/24/items/1.0166799

[11] Ti L, Tzemis D, Buxton JA (2012). Engaging people who use drugs in policy and program development: a review of the literature. Subst Abuse Treat Prev Policy.

[12] Greer A, Amlani A, Burgess H, Newman C, Burmeister C, Lampkin H, et al. Peer engagement principles and best practices for BC health authorities and other providers [internet]. BC Centre for disease control; 2018. Available from: http://www.bccdc.ca/health-professionals/clinical-resources/harm-reduction/peer-engagement-evaluation. Accessed 30 June 2018.

[13] “Nothing About Us Without Us” Greater, Meaningful Involvement of People Who Use Illegal Drugs: A Public Health, Ethical, and Human Rights Imperative [Internet]. Canadian HIV/AIDS Legal Network; 2006 Mar. Available from: http://www.aidslaw.ca/site/wp-content/uploads/2013/04/Greater+Involvement+-+Bklt+-+Drug+Policy+-+ENG.pdf. Accessed 30 June 2018.

[14] Greer A, Luchenski SA, Amlani AA, Lacroix K, Burmeister C, Buxton JA. Peer engagement in harm reduction strategies and services: a critical case study and evaluation framework from British Columbia, Canada. BMC Public Health [Internet]. 2016 [cited 2016 Jul 8];16. Available from: http://bmcpublichealth.biomedcentral.com/articles/10.1186/s12889-016-3136-4 10.1186/s12889-016-3136-4 PMC4882818 27229314

[15] Peer Engagement and Evaluation Project (PEEP) [Internet]. Vancouver, BC: BC Centre for Disease Control; 2016. Available from: http://www.bccdc.ca/our-services/programs/peer-engagement. Accessed 30 June 2018.

[16] Bogdan R, Biklen S (1982). Qualitative research for education: an introduction to theory and methods.

[17] Peer Engagement and Evaluation Project (PEEP) [Internet]. BC Center for Disease Control; 2017. Available from: http://www.bccdc.ca/resource-gallery/Documents/PEEP%20infographic%20v08%5b1%5d.pdf. Accessed 30 June 2018.

[18] Compassionate Engagement Modules [Internet]. Vancouver, BC: BC Center for Disease Control; 2017. Available from: http://www.towardtheheart.com/reducing-stigma. Accessed 30 June 2018.

[19] Minkler M (2010). Linking science and policy through community-based participatory research to study and address health disparities. Am J Public Health.

[20] Guta A, Flicker S, Roche B (2013). Governing through community allegiance: a qualitative examination of peer research in community-based participatory research. Crit Public Health.

[21] Greer A, Buxton J (2018). A guide for paying peer research assistants: challenges and opportunities (version 2) [Internet].

[22] Mackenzie S, Pearson C, Frye V, Gómez CA, Latka MH, Purcell DW (2012). Agents of change: peer mentorship as HIV prevention among HIV-positive injection drug users. Subst Use Misuse..

[23] Pyett P (2002). Working together to reduce health inequalities reflections on a collaborative participatory approach to health research. Aust N Z J Public Health.

[24] Langan D, Morton M (2009). Reflecting on community/academic ‘collaboration’ the challenge of ‘doing’ feminist participatory action research. Action Res.

[25] Room R (2005). Stigma, social inequality and alcohol and drug use. Drug Alcohol Rev.

[26] Belle-Isle L. At the table with people who use drugs: transforming power inequities [Internet] [Thesis]. 2016 [cited 30 Jun 2018]. Available from: https://dspace.library.uvic.ca//handle/1828/7199. Accessed 30 June 2018.

[27] Lancaster K, Santana L, Madden A, Ritter A (2015). Stigma and subjectivities: examining the textured relationship between lived experience and opinions about drug policy among people who inject drugs. Drugs Educ Prev Policy..

[28] Pitts M, Smith A. Researching the margins: strategies for ethical and rigorous research with marginalised communities. United Kingdom: Palgrave Macmillan; 2007.

[29] Souleymanov R, Kuzmanović D, Marshall Z, Scheim AI, Mikiki M, Worthington C, et al. The ethics of community-based research with people who use drugs: results of a scoping review. BMC Med Ethics [Internet]. 2016 [cited 30 Jun 2018];17. Available from: https://bmcmedethics.biomedcentral.com/articles/10.1186/s12910-016-0108-2 10.1186/s12910-016-0108-2 PMC4850694 27129927

[30] Marshall Z, Dechman MK, Minichiello A, Alcock L, Harris GE (2015). Peering into the literature: a systematic review of the roles of people who inject drugs in harm reduction initiatives. Drug Alcohol Depend.

[31] Damon W, Callon C, Wiebe L, Small W, Kerr T, McNeil R (2017). Community-based participatory research in a heavily researched inner city neighbourhood: perspectives of people who use drugs on their experiences as peer researchers. Soc Sci Med.

[32] Lazarus L, Shaw A, LeBlanc S, Martin A, Marshall Z, Weersink K (2014). Establishing a community-based participatory research partnership among people who use drugs in Ottawa: the PROUD cohort study. Harm Reduct J.

[33] Hayashi K, Fairbairn N, Suwannawong P, Kaplan K, Wood E, Kerr T (2012). Collective empowerment while creating knowledge: a description of a community-based participatory research project with drug users in Bangkok, Thailand. Subst Use Misuse..

[34] Savage CL, Xu Y, Lee R, Rose BL, Kappesser M, Anthony JS (2006). A case study in the use of community-based participatory research in public health nursing. Public Health Nurs.

[35] Blumenthal DS (2011). Is community-based participatory research possible. Am J Prev Med.

[36] McCall J, Mollison A, Browne A, Parker J, Pauly B (2017). The role of knowledge brokers: lessons from a community based research study of cultural safety in relation to people who use drugs. Can J Action Res.

[37] Newman SD, Andrews JO, Magwood GS, Jenkins C, Cox MJ, Williamson DC. Community Advisory Boards in Community-Based Participatory Research: A Synthesis of Best Processes. Prev Chronic Dis. 2011;A70. PMC3103575 21477510

[38] The involvement of drug user organisations in Australian drug policy: a research report from AIVL’s “Trackmarks” project [Internet]. Australian Injecting and Illicit Drug Users League (AIVL); 2012 Nov. Available from: https://nuaa.org.au/wp-content/uploads/2016/11/T4.7.2-aivl-drug-user.pdf. Accessed 30 June 2018.

[39] Treloar C, Fraser S, Valentine K (2007). Valuing methadone takeaway doses: the contribution of service-user perspectives to policy and practice. Drugs Educ Prev Policy.

[40] Lancaster K, Sutherland R, Ritter A (2014). Examining the opinions of people who use drugs towards drug policy in Australia. Drugs Educ Prev Policy..

[41] Hayashi K, Small W, Csete J, Hattirat S, Kerr T. Experiences with Policing among People Who Inject Drugs in Bangkok, Thailand: A Qualitative Study. PLoS Med [Internet]. 2013 [cited 30 Jun 2018];10. Available from: http://journals.plos.org/plosmedicine/article/comments?id=10.1371/journal.pmed.1001570. Accessed 30 June 2018. 10.1371/journal.pmed.1001570 PMC3858231 24339753

[42] Stewart KE, Wright PB, Sims D, Tyner KR, Montgomery BEE (2012). The “translators”: engaging former drug users as key research staff to design and implement a risk reduction program for rural cocaine users. Subst Use Misuse.

[43] Coser LR, Tozer K, Van Borek N, Tzemis D, Taylor D, Saewyc E (2014). Finding a voice: participatory research with street-involved youth in the youth injection prevention project. Health Promot Pract.

